# About Non-Line-Of-Sight Satellite Detection and Exclusion in a 3D Map-Aided Localization Algorithm

**DOI:** 10.3390/s130100829

**Published:** 2013-01-11

**Authors:** Sébastien Peyraud, David Bétaille, Stéphane Renault, Miguel Ortiz, Florian Mougel, Dominique Meizel, François Peyret

**Affiliations:** 1 XLIM, UMR CNRS 7252, Limoges University, 87060 Limoges, France; E-Mails: renault@ensil.unilim.fr (S.R.); dominique.meizel@xlim.fr (D.M.); 2 French Institute of Science and Technology for Transport, Development and Networks (IFSTTAR), 44344 Bouguenais (Nantes), France; E-Mails: david.betaille@ifsttar.fr (D.B.); miguel.ortiz@ifsttar.fr (M.O.); florian.mougel@ifsttar.fr (F.M.); francois.peyret@ifsttar.fr (FP.)

**Keywords:** localization, satellite navigation, intelligent transportation system, NLOS, 3D navigable road maps, 3D kinematic model, data fusion, EKF, set membership estimation

## Abstract

Reliable GPS positioning in city environment is a key issue actually, signals are prone to multipath, with poor satellite geometry in many streets. Using a 3D urban model to forecast satellite visibility in urban contexts in order to improve GPS localization is the main topic of the present article. A virtual image processing that detects and eliminates possible faulty measurements is the core of this method. This image is generated using the position estimated a priori by the navigation process itself, under road constraints. This position is then updated by measurements to line-of-sight satellites only. This closed-loop real-time processing has shown very first promising full-scale test results.

## 1. Introduction

Reliable GPS positioning in city environment is a key issue: actually, signals are prone to multipath, and satellite geometry, despite its improvement with GNSS interoperability, remains poor in many streets. Non-Line-Of-Sight (NLOS) satellites cause important receiver-satellite range measuring errors, because the direct signal is blocked and only the reflected signal is tracked. Contrary to multipath where direct and reflected signals interfere, errors with NLOS satellites grow unboundedly. The geometrical properties of the local environment of the antenna can explain deterministically the phenomena that occur, which makes 3D city models of great interest in this tricky problem.

First, let us mention, even if there is no use of 3D models, the image processing approach like, e.g., that of the LocoPROL project (Low cost satellite based train location system for signalling and train PROtection for Low density railway lines) [1]. This approach already uses an obstacle elevation model, characterized from a fish-eye camera for both sides of a railway, in order to determine whether a received signal should be considered or not, in which case the corresponding satellite turns out to be masked from the user.

Concerning 3D models, in 2007, Bradbury *et al.* [2] have investigated the possibility of using building description in the vicinity of the antenna in order to mitigate multipath and occlusion. Suh and Shibasaki [3] also make use of 3D data bases to predict GNSS quality of service.

Well-founded results were shown with these first contributions, but more recently, a full-scale experiment of this concept applied to localization in a 3D modelled urban center has been proposed in CityVIP [4]: a 2008–2011 project that aims at designing a global management system of a fleet of autonomous individual transportation vehicles. Concerning NLOS detection and city model, a preliminary proof of concept has recently been published in IEEE Intelligent Transport System Telecommunications (ITST) 2011 [5]. In this article, the position from which the 3D model is considered in order to compute the critical elevations at satellite azimuths was delivered by a high-grade kinematic GPS and inertial navigation system. The success of the demonstration using, for satellites visibility, the off-line “true” position of the vehicle confirmed the relevance of the concept. However, this could not lead us to predict whether or not the idea would work when one uses a standard GPS position in place of an accurate reference point for the computation of the satellite critical elevation. This is the aim of the present article.

Similar approaches are proposed by [6,7], with more simple map models, and by [8], with ray-tracing algorithm. Last but not least, let us mention the approach that was proposed by [9,10], under the name “shadow matching”: the investigations undertaken aim at testing which one from a set of possible localizations around a standard initial solution is the most probable with respect to the coherence between a 3D model based prediction of LOS/NLOS satellites and the actual satellites in view. First results in London are very promising in terms of street lane separation including sidewalk. Additionally, shadow matching appears complementary to direct NLOS detection methods, as presented by [11].

This article is divided into three main sections. We first explain the methodology and mention the results obtained in the feasibility study based on a kinematic GPS INS solution. In the second part, a map-aided positioning filter is presented that takes advantage of the information given by the 3D model in terms of road constraints. The third and last part analyzes the experimental results obtained by the LOS/NLOS separation algorithm based on the previous constrained solution. The efficiency of the road constraints in this process will be demonstrated. Finally, we will discuss the impact of the separation algorithm and its interest with respect to the localization problem, before and after a final map-aiding, and we will compare this algorithm to a more usual satellite selection based on signal-to-noise ratio (SNR). These comparisons are done from data registered in a real experiment in the center of Paris (France). The duration of this experiment is about 10 minutes and the total length of the circuit is about 2 km in an archetypal European urban center.

## 2. Presentation of the NLOS Detection Method

**Figure 1.**
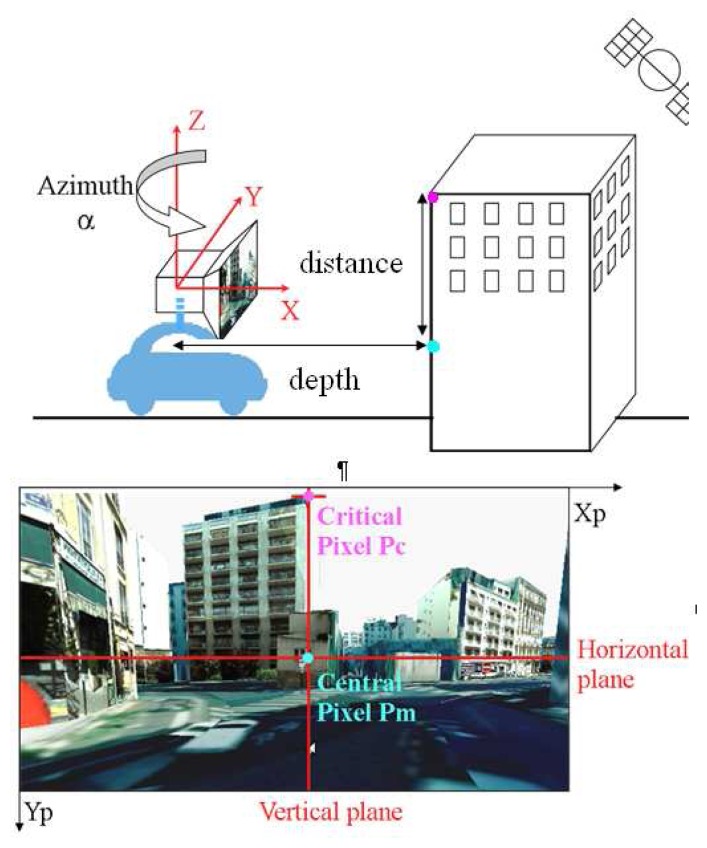
Illustration of the computation of the critical elevation using a virtual image.

The core of the method consists in checking whether a satellite that generates a signal received by the GNSS receiver can be directly viewed (LOS) or not (NLOS) by the receiver antenna. With this aim in mind, one needs the predicted position of the receiver, the predicted positions of the satellites, and the map of the environment in the same reference frame. A 3D city model of the environment is available in a Geographical Information System (GIS) embedded software from BeNomad company [12], SIVNav SDK (Software Development Kit), which is dedicated to augmented reality, 3D map rendering and vision for robotics. It contains a geometrical description of buildings, roads (actually streets) and sidewalks. The 3D elements that are in the vicinity of the receiver localization are extracted from the database, and a virtual image is returned. Extraction and image computation are basic functions of the GIS. The availability of this software, on the CityVIP platform, has motivated the use of a virtual image. Geographical data are provided by the French National Geographical Institute (IGN), in the national French plane projection (Lambert 93) plus mean sea level (MSL) altitude (further called: the GIS reference frame). Their accuracy is: 5 cm horizontally and 25 cm vertically in Paris [13,14],

The method starts with the computation of a virtual image of each satellite, with a virtual camera located at the antenna center, oriented with the azimuth of the considered satellite, with tilt angles (roll and pitch) set to zero (Figure 1). An important parameter of the virtual camera is its focal distance. From an initial value, this is iteratively reduced until the sky is visible on top of the frontal building. The sky visibility may not be obtained in case this building would be very close to the user, which entails NLOS for the corresponding satellite.

Basic image processing functions provided by BeNomad make it possible to compute the front building elevation. These functions are twofold: Get_depth (pixel), which returns the depth of the closest point corresponding to the input pixel in the 3D model of the environment, Get_distance (pixel_1, pixel_2), which returns the Euclidian distance between the closest points of the 3D model that corresponds to pixel_1 and pixel_2. The geometrical computation of the critical elevation *β_c_* (1) by using the output of these functions applied onto the central and critical pixels respectively is illustrated on Figure 1.

The comparison of the satellite elevation *β* to this threshold makes the final decision on whether the satellite is considered NLOS or not.

(1) $$\beta_{c}=\text{atan}(\frac{\text{Get}\_\text{distance}(P_{c},P_{m})}{\text{Get}\_\text{depth}(P_{m})})$$

A more straightforward method consists in computing the virtual image with the camera tilted according to the elevation of the satellite, and, like ray tracing, check whether or not a pixel is detected along the optical axis (if not, Get_depth (pixel) returns −1 and the satellite is in LOS). The standard focal distance is always suitable. Note that the critical elevation is no more available that way, but actually not essential.

In practice, azimuth and elevation of satellites are delivered by standard NMEA (National Marine Electronics Association) GSV (Satellites in view) messages. Note that the correction of the azimuthal deviation (up to a few degrees) between the true north and the north of the map (the one using the Lambert 93 plane projection) must be done.

**Figure 2.**
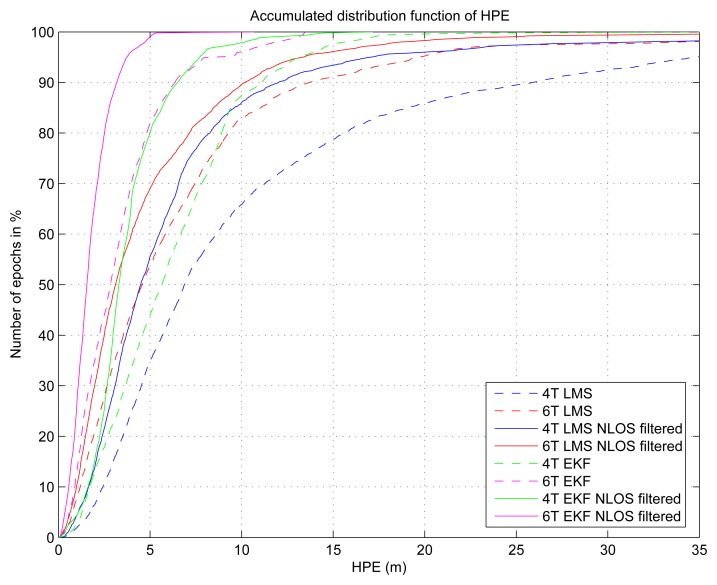
Cumulative distribution function of the horizontal position error (HPE).

The position of the user is in fact the most critical point in the process. In a first step of this research [5], we used our Reference Trajectory Measurement (MRT) [15] system to produce the accurate position that feeds our NLOS algorithm. The results we obtained and published were very promising (Figure 2), but there was no error on the estimation of the position of the user that might bring an error in the NLOS detection itself, with the risk of a possible divergence.

Here, the position of the antenna will come from a City VIP positioning sub-system that combines data from dead-reckoning sensors and GNSS, under the 3D map constraints.

Figure 2 shows the impact of ground-truth-based NLOS detection and exclusion (for 2 receivers U-blox LEA-4T and LEA-6T and for both least square (LS) and extended Kalman filter (EKF) solutions): solid lines (corresponding to NLOS filtered solutions) are always closer to the y-axis than dashed lines (non-filtered solutions), which proves the efficiency of the LOS/NLOS separation algorithm.

## 3. Presentation of the Positioning Method with Road Constraints

The method consists in computing a 3D localization of a wheeled vehicle based on a 3D kinematic model and improved by map-aiding on the road layer of the 3D city map. This functionality was a task of the CityVIP project [4].

A state space formulation of the problem is used in order to use state estimation techniques such as variants of Kalman filtering. As it is usual in Robotics, the configuration is taken as a state vector. The kinematic model then gives the progression part of the state equation.

### 3.1. Definition of the Vehicle Configuration (Figure 3)

**Figure 3.**
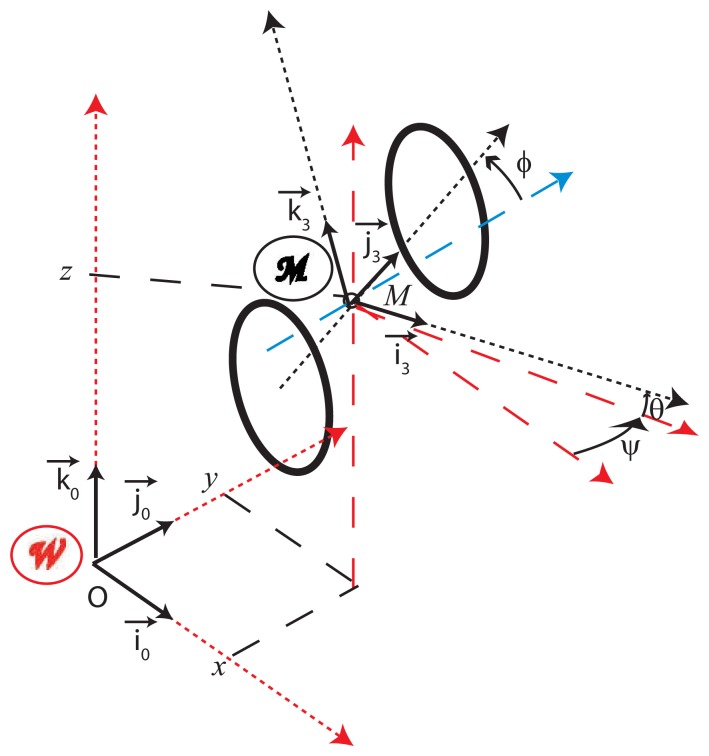
3D vehicle configuration for a fixed rear axle model, with the world and mobile reference frames as 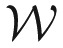 and 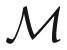 respectively.

The world reference frame being the one where the travel is specified (*i.e.*, the GIS reference plane in Lambert 93 coordinates), let us denote 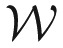: (*O*, *i⃗*_0_, *j⃗*_0_, *k⃗*_0_) as this world reference frame and 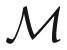: (*M*, *i⃗*_3_, *j⃗*_3_, *k⃗*_3_) as the vehicle reference frame (Figure 3). By definition, the vehicle configuration states the pose of the vehicle reference frame with respect to the world frame. In the 3D Euclidian world, it may be defined by:

(2) $$\begin{array}{c} q={\left[x,y,z,\psi ,\theta ,\phi \right]}^{T}\\ \text{with}:\{\begin{array}{l} \psi :\text{heading},\\ \theta :\text{slope},\\ \phi :\text{cross slope} \end{array}\text{and}\;(x,y,z)\;\text{the}\;3D\;\text{coordinates of the middle of the rear axle}\;[16]. \end{array}$$

In other words, the configuration may be defined as the transformation from W to 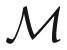 by 4 elementary operations: one translation (*OM⃗*) and 3 successive rotations (*ψ*, *θ*, *φ*). Note that one considers here a vehicle without suspension. The (*ψ*, *θ*, *φ*) orientation angles are thus only induced by the geometry of the road and the path followed by the vehicle.

### 3.2. Kinematic Model Processing

The rationale of the model is based upon the motion of the wheels that roll without slipping on a plane surface. Here the plane is inclined and its inclination is assumed to evolve slower than the other variables (in particular, slope and cross slope angles have slower variations than the one of the yaw angle). Note that such a model is coherent with the 3D map composed of planar patches that is further used.

Moreover, like in the usual 2D kinematic models, the longitudinal speed (*υ*) and the yaw angular rate (*ω*) in the evolution plane are assumed to be known from odometry measurements.

For a vehicle with two motorized wheels where:

○ *E* denotes the track, e.g., the distance between the centers of the left and right wheels,
○ *ω_r_* (resp. *ω_l_*) is the measured rotation speed of the right (resp. left) wheel,
○ *R_r_* (resp. *R_l_*) is the radius of the right (resp. left) wheel, assumed to be known.

by measuring the rotation speed one gets the relation (3)

(3) $$\left[\begin{array}{c} \upsilon \\ \omega \end{array}\right]=\left[\begin{array}{cc} \frac{1}{2} & \frac{1}{2}\\ \frac{1}{E} & -\frac{1}{E} \end{array}\right]\cdot \left[\begin{array}{c} R_{r\cdot }\omega_{r}\\ R_{l\cdot }\omega_{l} \end{array}\right]$$

From the non-holonomic constraints linked to the rolling without slipping rotations of the wheels on the plane surface, the vector speed of *M* is aligned with the axis *i⃗*_3_ of the vehicle (Figure 3) and its norm is *υ* given by Equation (3). In the same way, the axis of the yaw rotation is the local normal *k⃗*_3_ to the road and its norm is *ω* given by Equation (3).

Expressing those relations in the world reference frame (*i.e.*, the GIS reference plane in Lambert 93 coordinates) gives the global kinematic state Equation (4) [17]. This model appears to have common features with a simplified dynamical model [18].

(4) $$\left[\dot{q}\right]=G(q)\cdot \left[\begin{array}{c} \upsilon \\ \omega \end{array}\right];G(q)=\left[\begin{array}{cc} \cos\psi \cdot \cos\theta & 0\\ \sin\psi \cdot \sin\theta & 0\\ -\sin\theta & 0\\ 0 & \frac{\cos\phi }{\cos\theta }\\ 0 & -\sin\phi \\ 0 & \tan\theta \cdot \cos\phi \end{array}\right]$$

A discrete time version (5) is deduced from Equation (4) by the Euler formula for a real-time implementation

(5) $$q_{k+1}=q_{k}+G(q_{k})\cdot \left[\begin{array}{c} ds_{k}\\ d\psi_{k} \end{array}\right]$$

In this expression, *ds_k_* = *υ_k_* * (*t_k_*_+1_ − *t_k_*) (resp. *dψ_k_* = *ω_k_* * (*t_k_*_+1_ − *t_k_*)) is the elementary travelled distance (resp. elementary yaw rotation in 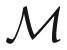) between the successive time samples *t_k_* and *t_k_*_+1_.

### 3.3. Localization Method

**Figure 4.**
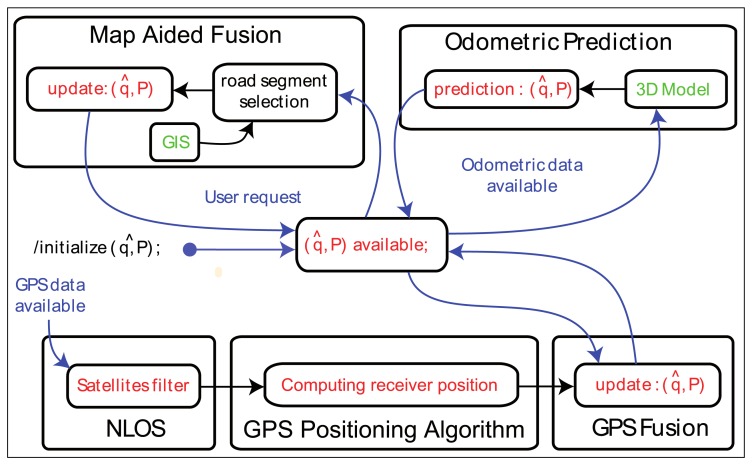
Data fusion state chart.

In the CityVIP project, a localization task has been introduced in order to continuously update an estimation of the configuration of the vehicle in a 3D world together with an accuracy statement. This estimation is iteratively computed by combining 3 data sources (Figure 4), each one with a different time scale:

1. GPS localization is obtained by a positioning algorithm at the update rate of the receiver (4 Hz). A complete localization may be scarce in environments with poor satellite visibility
2. odometric data are generated by the wheel speed sensors of the ABS (Anti-Blocking System). When some data become available, the configuration is updated by using Equation (5),
3. geographic data, 3D polylines modelling the road network, are given upon request but require a map-matching procedure prior to using them for localization.

In this research, an Extended Kalman Filtering (EKF) is used in the odometric/GPS fusion process. Geographic data are used to constrain the localization with an ellipsoidal set-membership method [19]. At any time *t_k_* where some information becomes available, the algorithm updates an estimate *q̂_k_* together with a symmetric positive matrix *P_k_*, defining thus an ellipsoidal confidence domain [20]:

(6) $${\left(q-\hat{q_{k}}\right)}^{T}\cdot P_{k}^{-1}\cdot (q-\hat{q_{k}})<1$$

Note that the symmetric positive matrix *P_k_* quantifies the magnitude of the ellipsoidal domain. The square-roots of its eigenvalues are the measures of its principal axes.

#### Odometric and GPS Data Fusion

Each time odometric measurements are available (Figure 4), a new prediction is performed by using the state propagation Equation (5). If GPS data are also available, an a posteriori update is realized by an EKF algorithm. The measurement noise covariance matrix is obtained from GPS inaccuracy and the state noise covariance matrix is deduced from the propagation of individual inaccuracies [21]. These noises are assumed to be Gaussian, unbiased, white with a priori known covariance matrices.

This gives an updated estimation *q̂_k_* and an updated matrix *P_k_* resulting from both odometric and GPS measurements, when available.

#### Map-Aided Fusion

The use of the geographical data is based on the assumption that the vehicle moves on a road with a known geometric specification stored in the GIS. It is a sequence of two steps:

- Map-matching, i.e., the selection of the road segment on which the vehicle is supposed to be. The segment should minimize a criterion calculated from (1) the 3D distance between the current estimation of the localization and the segment and (2) the angular error between the velocity vector of the state and direction of the segment.
- Exploitation of the geometric attributes of the road segment selected as constraints of the configuration (2). Constraints are defined from the 3D polylines by taking into account the width of the way and the uncertainty on the altitude. The ellipsoidal set-membership method computes the minimum volume ellipsoid resulting from the intersection of the current ellipsoidal domain and constraints. The final map-aided solution is obtained [22].

## 4. Improving the NLOS Detection Method and Experimental Results

### 4.1. Experimental Set-Up and Test Data

For validation purpose, we use data that have been recorded in an experiment carried out for the final demonstration of the cityVIP project in September 2011 in Paris. The data were collected on 13 September 2011, and two circuits, both with two laps, were performed at respectively 11:48 AM and 12:08 PM (local time). The first lap was used by the CityVIP partners in charge of several image processing localization tasks for their machine learning; afterwards, the second and next laps could be used for evaluation purpose.

The distance travelled per lap is around 1 kilometer. The urban environment in the vicinity of the 12*^th^* district city hall is very dense, with high Haussmann style buildings.

**Figure 5.**
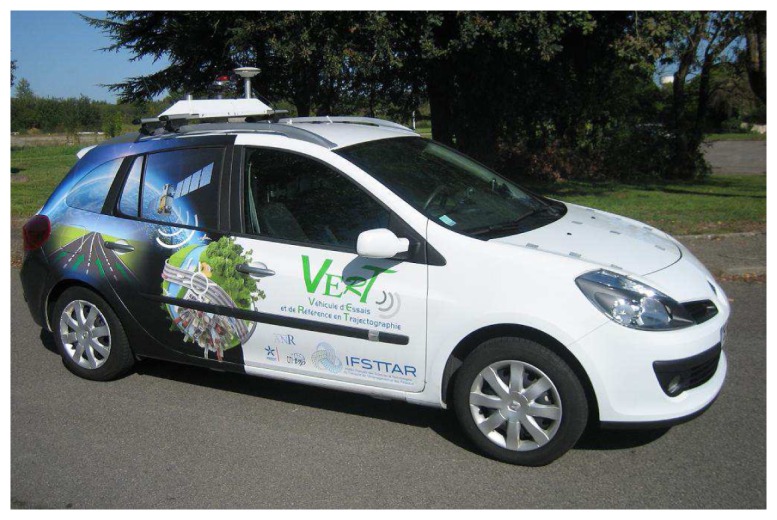
The experimental vehicle of IFSTTAR/MACS.

The experimental vehicle of IFSTTAR/MACS (Monitoring, Assessment, Computational Sciences) is shown in Figure 5. It has been used for the final demonstration of the CityVIP project in September 2011 and was equipped with:

- a CAN (Controller Area Network) bus connection (for the odometry),
- a low-cost automotive GPS receiver LEA-6T from U-blox (for raw data and NMEA GGA [23], Global Positioning System Fix Data, and GSV, Satellites in view, sequences at 4 Hz) and its patch antenna,
- the MRT (Reference Trajectory Measurement) dedicated specific equipment, LANDINS of the IXSEA society, from which the reference trajectory of the present experiment is issued. Its accuracy is about 10 centimeters,
- a Marlin video camera (not used here).

Data were logged in real-time using the Aroccam multithread software architecture [24] and processed off-line in “replay” mode. The minimum system requirements to perform these tests are: a multi-core processor (quad core at 2.4 GHz used here), 2 GB of RAM and a basic video card (1 GB Nvidia Quadro FX 2880 M used here).

### 4.2. NLOS Detection Based on Map-Aided Solutions

Typical errors of standard GPS solutions are so large (several tens of meters) that creating a virtual image would be nonsense. As a consequence, the image extraction is based on the output of the map-aided positioning process where road constraints have been applied. To do so, the “Map-Aided Fusion” task of the state chart (Figure 4) has been duplicated, and the duplicated process is, at this stage, fed with the NMEA GGA solutions where the data of the satellites are used irrespective of their LOS/NLOS status and coupled with the odometry data.

Afterwards, two different subsequent processes are possible:

- The LOS satellites are only fed to the GPS Positioning Algorithm and fused with the odometry. It yields non-map-aided or “free” solutions. In this case, we collect the updated position and covariance of the “GPS Fusion” task.
- The LOS satellites are only fed to the GPS Positioning Algorithm, fused with the odometry, and constrained again by the road map. It yields map-aided or constrained solutions and we collect the updated position and covariance of the “Map-Aided Fusion” task. This very last implementation can run in closed-loop, since its solution can be returned to the image extractor, which makes the duplication of the “Map-Aided Fusion” task not necessary.

### 4.3. Comparison with the SNR-Based Selection

The purpose of this final section is to evaluate whether or not the LOS/NLOS separation leads to positions at least as accurate as a simple SNR-based selection would do.

The comparison has been made on one lap, *i.e.*, by using 5 minutes of data. A priori, with our GPS receiver at 4 Hz, 1,200 GPS epochs are available, with up to 12 satellites available per epoch, depending on the sky visibility. A few epochs have no visible satellite.

Four replications (in Aroccam replay mode) have successively been executed:

○ 1 - SNR-based satellite selection, no final map-aiding,
○ 2 - LOS-based satellite selection, no final map-aiding,
○ 3 - SNR-based satellite selection, and final map-aiding,
○ 4 - LOS-based satellite selection, and final map-aiding.

Note that the last replication (item 4) has been operated in closed-loop, whereas the second (item 2) used GGA map-aided solutions.

**Figure 6.**
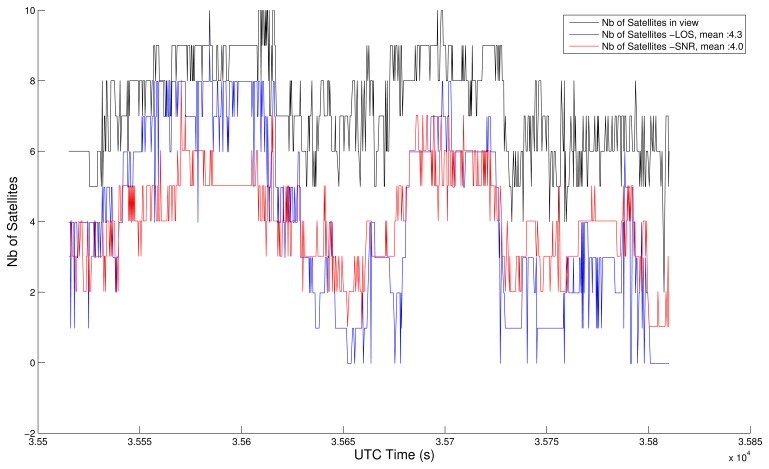
Satellite visibility using GGA map-aided solutions.

**Figure 7.**
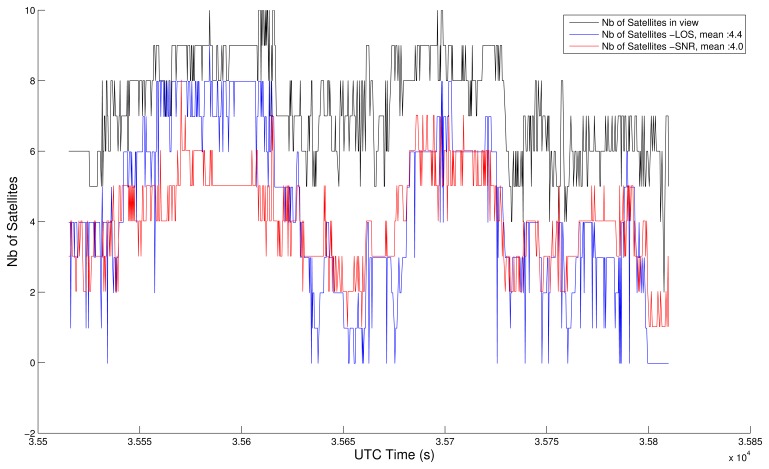
Satellite visibility in closed-loop.

The main tuning to do is the SNR threshold applicable to the GPS Positioning Algorithm. We have adjusted its value (40 dB-Hz) so that the average number of satellites actually used by both processes (SNR and LOS) are similar. Figures 6 and 7 show the satellite visibility corresponding to both tests, using GGA map-aided solutions (items 1 and 2) and in closed-loop (items 3 and 4). The SNR test is always the same, but the common epochs vary, which explains the slight differences in the number of satellites time series for SNR items. The NLOS test is not repeated strictly the same way, since the position from where the image is extracted varies. Anyway, both strategies (GGA map-aided or closed-loop) are coherent. The average number of satellites used during this 5 minutes test is around 4, with 40 dB-Hz threshold or NLOS detection and exclusion.

Statistics have been computed on the whole dataset, *i.e.*, on every common epoch.

**Figure 8.**
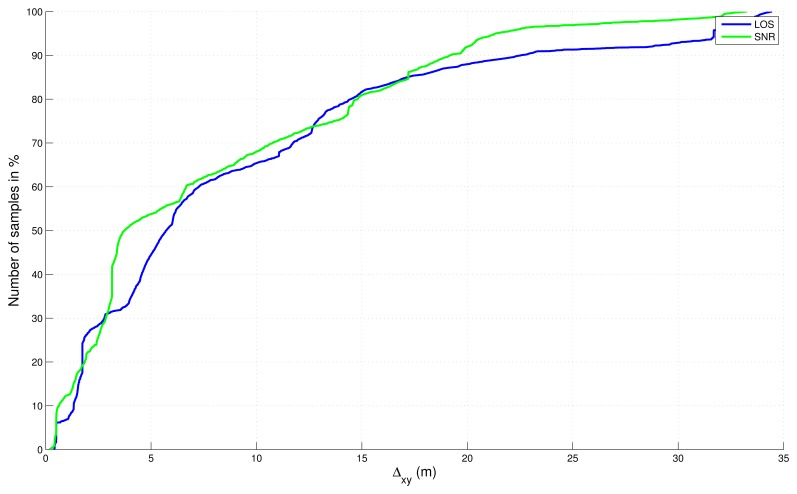
Cumulative distribution function of the absolute error in 2D for “free” solutions (without map-aiding).

**Figure 9.**
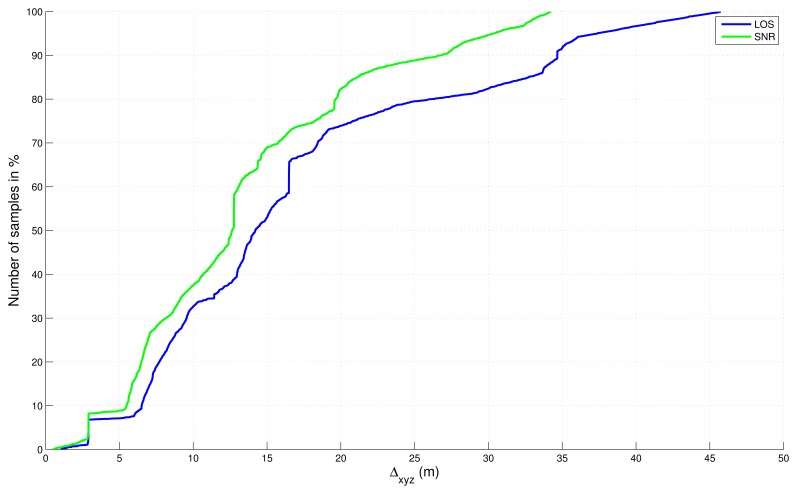
Cumulative distribution function of the absolute error in 3D for “free” solutions (without map-aiding).

**Figure 10.**
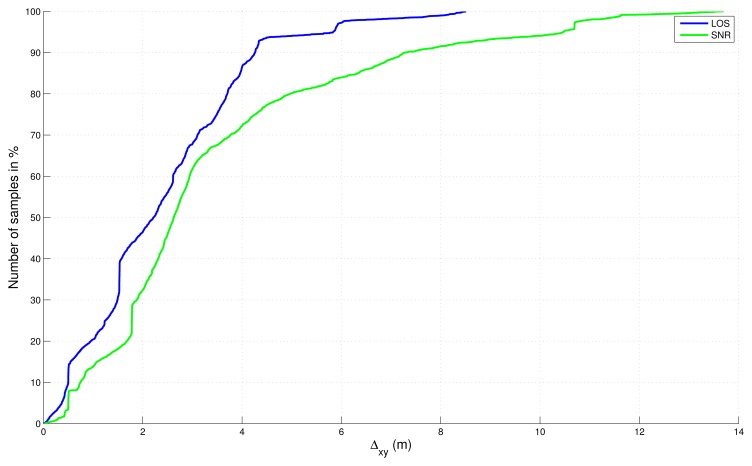
Cumulative distribution function of the absolute error in 2D for map-aided solutions.

**Figure 11.**
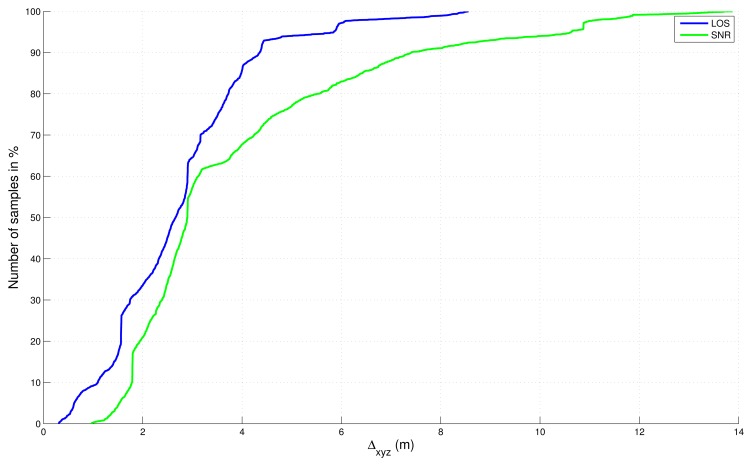
Cumulative distribution function of the absolute error in 3D for map-aided solutions.

Absolute errors, in 2D and 3D, with respect to the reference trajectory have been computed and sorted in a cumulative distribution function in Figures 8 and 9 without final map-aiding and in Figures 10 and 11 with final map-aiding.

The reference trajectory (provided by the LANDINS system) is the fusion result of an INS (Inertial Navigation System) based on a high-grade fiber optic gyroscope (FOG), a distance measurement instrument and a PPK (Post Processed Kinematic) trajectory from a high-performance GNSS receiver.

Considering the errors of the “free” solutions (Figures 8 and 9), it appears that the LOS filter is equivalent to the SNR filter for the horizontal dimension. Meanwhile, when the vertical dimension is also considered, SNR filter seems to be better. This result can be explained by the fact that our circuit get at the borders of the 3D model currently available. Outside these borders, indeed, neither trees nor buildings are modelled and the NLOS test is performed obstacle-free, which potentially causes satellites not in direct view to be considered as LOS. This is the case in the northwest area and the south area of our map, which is more penalizing when the vertical dimension is considered.

For map-aided solutions (Figures 10 and 11), conclusions are different in particular because map-aiding enables to constraint the vertical dimension: errors in 2D and 3D are very similar, which shows that the vertical dimension is well estimated. In this case, results are globally better than the “free” solutions and the result of the fusion is even slightly better with the LOS filter.

**Figure 12.**
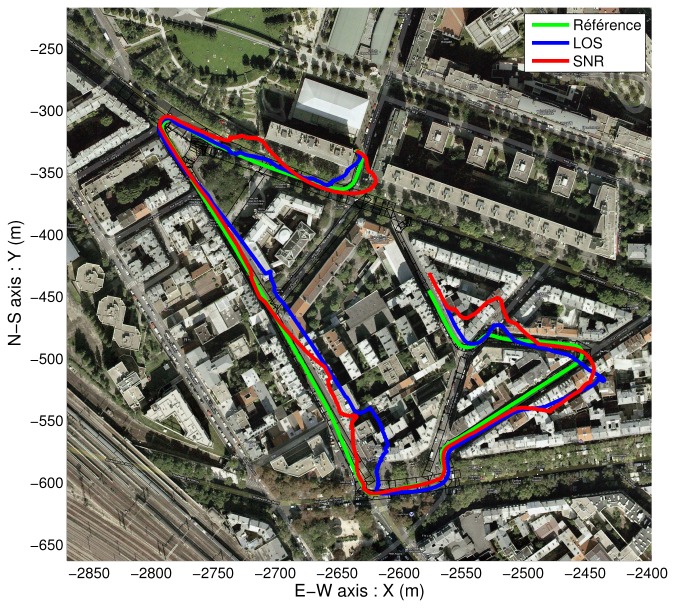
2D overview of “free” solutions (without map-aiding) for both strategies.

**Figure 13.**
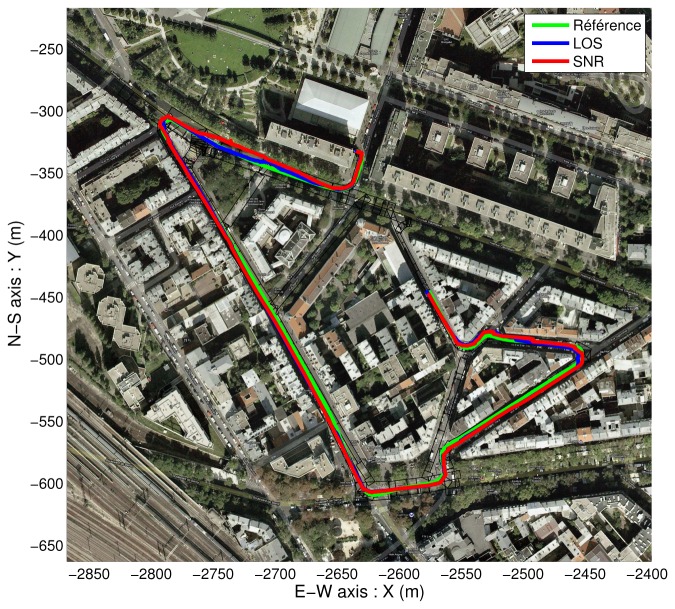
2D overview of map-aided solutions for both strategies.

An overview of the trajectories is visible in Figures 12 and 13 respectively.

**Figure 14.**
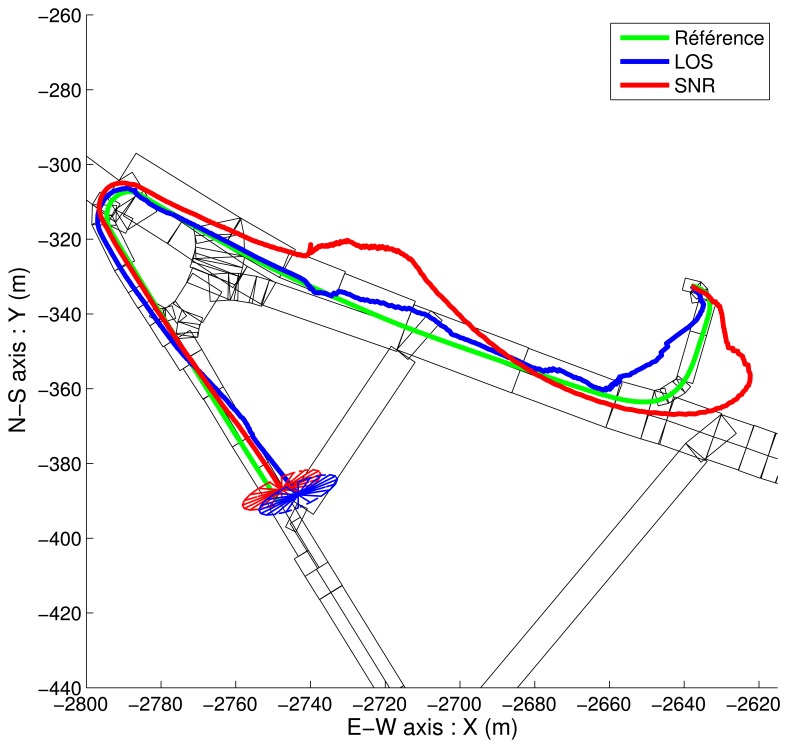
2D overview of “free” solutions (without map-aiding) for both strategies. Simplified representation.

**Figure 15.**
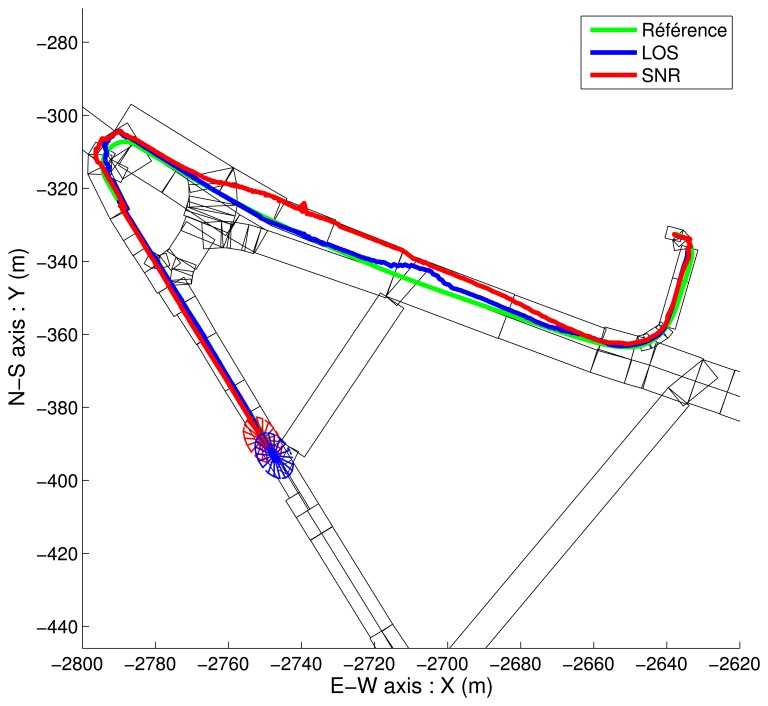
2D overview of map-aided solutions for both strategies. Simplified representation.

Since Figures 12 and 13 are overloaded, simplified representations of the beginning of the estimated trajectories are shown in Figures 14 and 15. These results show the various estimations of the path together with the elements of the map.

On Figure 14, the displayed estimations of the path seem to be non-admissible because they are often outside of the map. These estimations correspond punctually to the localization of the center of the admissible ellipsoidal domain (6) that always intersects at least one rectangular element of the map. The estimated solutions are thus admissible although not accurate. The accuracy of the estimation is illustrated by the projection of the admissible ellipsoidal domain onto the (*x*, *y*) plane for the last estimated point.

On Figure 15, the final map-aiding enhances the accuracy: the admissible ellipsoidal domain (its projection onto the (*x*, *y*) plane) for the last estimated point is, in both case, smaller. The constraints bound to the width of the road appear clearly. Both LOS and SNR based filtering give comparable results. These results also show the prior importance of the map-aiding.

## 5. Conclusions and Future Works

### 5.1. Conclusions

This paper has shown that using NLOS detection and exclusion prior to incorporating data from a GNSS receiver was efficient for the localization in dense urban areas. Such a test needs (1) 3D maps of the urban environment, which are more and more easily available and (2) an a priori estimation of the position and orientation of the vehicle.

The results have shown the prior importance of the 3D maps of the urban environment. First, it constrains the position and orientation estimations in coherence with the streets network, thus enhancing the accuracy of the localization. Moreover, it makes it possible to check the direct visibility of a given satellite from the estimated pose of the vehicle. The results shown in the paper lead to conclude that the NLOS test gives an equivalent accuracy to the SNR test when the estimated pose is not constrained by the map-aiding procedure after being updated by GNSS data (slightly worse when the vertical dimension is considered). In contrast, when the map-aiding procedure is applied after having used the GNSS data with the NLOS (or SNR) test, the NLOS test gives slightly better accuracy than the SNR test. It thus confirms the feasibility study previously exposed in [5] where the accuracy of the localization was significantly better.

NLOS detection and exclusion based on virtual image processing and a 3D map seems to be promising. This alternative to a simple SNR test on the satellites tracked by a standard automotive receiver has yielded better positioning.

### 5.2. Future Work

Given that this dataset corresponds to a relatively short experimentation (5 min, 1 km in Paris), other tests will be realized to confirm our first conclusions.

The GPS Positioning Algorithm was also very simple: no fault detection or exclusion of satellites (FDE strategy) based on the predicted position (and consequently predicted pseudo-ranges) was done in addition to the SNR or LOS tests. A future version of our algorithm will improve this Positioning Algorithm, including FDE through a *χ*^2^ test. Doppler measurements, which are not employed in the current study, will also be used.
